# Sodium citrate pretreatment enhances CAR-T cell persistence and anti-tumor efficacy through inhibition of calcium signaling

**DOI:** 10.3389/fimmu.2025.1540754

**Published:** 2025-03-17

**Authors:** Xuechen Yin, Wenwen Chen, Xudong Ao, Luxia Xu, Jiujiu Cao, Tinghui Huang, Junqing Liang, Jianhua Hu, Jiaqi Liu, Xinping Wang, Wenying Li, Muya Zhou, Lingfeng He, Zhigang Guo

**Affiliations:** ^1^ Jiangsu Key Laboratory for Molecular and Medical Biotechnology, College of Life Sciences, Nanjing Normal University, Nanjing, China; ^2^ Peking University Cancer Hospital (Inner Mongolia Campus)/Affiliated Cancer Hospital of Inner Mongolia Medical University, Hohhot, China; ^3^ Center of Biotherapy, Jiangsu Province Geriatric Hospital, Nanjing, China

**Keywords:** CAR-T, T cell exhaustion, calcium ions, sodium citrate, cancer immunotherapy, solid tumors

## Abstract

**Introduction:**

Chimeric antigen receptor T cell (CAR-T) therapy has shown success in treating hematological malignancies, but its effectiveness against solid tumors is hindered by T cell exhaustion. During *in vitro* expansion, tonic signaling induced by CAR expression contributes to CAR-T cell exhaustion, which can be mitigated by inhibiting calcium signaling. Given that sodium citrate can chelate calcium ions and inhibit calcium signaling, in this study, we investigated whether sodium citrate could reduce exhaustion and enhance CAR-T cell function.

**Methods:**

We constructed anti-CD70 CAR-T cells and cultured them in the presence of sodium citrate. The characteristics and functionality of sodium citrate-pretreated CAR-T cells were assessed through *in vitro* and *in vivo* experiments. To further validate our observation, we also treated anti-mesothelin (MSLN) CAR-T cells with sodium citrate and detected the phenotypes and anti-tumor function of CAR-T cells.

**Results:**

We found that sodium citrate-pretreated anti-CD70 CAR-T cells exhibited reduced exhaustion, increased memory T cell proportions, and enhanced anti-tumor efficacy both *in vitro* and *in vivo*. Notably, sodium citrate treatment improved the *in vivo* persistence of CAR-T cells and prevented tumor recurrence. These beneficial effects were also observed in anti-MSLN CAR-T cells. Transcriptomic and metabolite analyses revealed that sodium citrate inhibited calcium signaling, mTORC1 activity, and glycolysis pathways, thus modulating T cell exhaustion and differentiation.

**Discussion:**

Our findings suggest that sodium citrate supplementation during CAR-T cell expansion could be a promising strategy to improve CAR-T therapy for solid tumors by preventing exhaustion and promoting memory T cell formation.

## 1 Introduction

Chimeric antigen receptor (CAR) T cells, or CAR-T cells, are genetically engineered T cells that express CARs on their surface, enabling them to directly recognize and bind to specific antigens, triggering T cell activation (1–3). CAR-T cell therapy has demonstrated remarkable success in treating hematological malignancies, including acute lymphoblastic leukemia (ALL), chronic B-lymphocytic leukemia (B-CLL), and lymphoma (4–7). However, its effectiveness in treating solid tumors has been more limited (8–10). A major obstacle to the success of CAR-T therapy in solid tumors is T cell exhaustion, a phenomenon that impairs the long-term function of CAR-T cells (11–14). T cell exhaustion was first identified in CD8+ T cells during chronic lymphocytic choriomeningitis virus (LCMV) infection and has since been recognized as a significant barrier to CAR-T efficacy, particularly in solid tumors (5, 15). Compared to hematological malignancies, solid tumors present a more challenging environment due to their immunosuppressive microenvironment, which accelerates T cell exhaustion (16–18). Overcoming T cell exhaustion is regarded as one of the effective ways to enhance anti-solid tumor activity of CAR-T cells.

T cell exhaustion is closely linked to the differentiation state of T cells (19). T cells can be classified into five stages based on their differentiation: naïve T cells, T stem cell-like memory (Tscm) cells, central memory T (Tcm) cells, effector memory T (Tem) cells, and effector T (Teff) cells. In CAR-T therapy, cells in the naïve, Tscm, Tcm, and Tem states are less prone to exhaustion than Teff cells, making the promotion of a memory-like phenotype in CAR-T cells critical for enhancing their persistence and long-term anti-tumor activity (20). Tonic signaling, which occurs in the absence of antigen stimulation, can drive terminal differentiation and exhaustion of CAR-T cells, limiting their effectiveness *in vivo*. Therefore, strategies to modulate the differentiation state of CAR-T cells and minimize tonic signaling are essential for improving CAR-T therapy outcomes in solid tumors (21–26).

Recent studies have explored the use of metabolic regulators, epigenetic modifiers, and pharmacological inhibitors to redirect CAR-T cell differentiation toward a memory-like state, thereby reducing exhaustion (27). As key second messengers, calcium ions (Ca^2+^) have been reported to play a pivotal role in inducing T cell exhaustion (23, 28, 29). Sodium citrate is a sodium salt of citric acid used in food and can function as a chelating agent to inhibit intracellular calcium signaling in various tumor cells (30, 31). Therefore, we hypothesized that through calcium inhibition, sodium citrate supplementation during CAR-T cell culture could inhibit tonic signaling, reduce exhaustion, and enhance the long-term anti-tumor activity of CAR-T cells.

To test this hypothesis, we constructed CD70-specific CAR-T cells, as CD70 is highly expressed in solid tumors (32, 33). Sodium citrate was added to the culture media, and the effects on CAR-T cell exhaustion and anti-tumor activity were assessed. Our results demonstrated that sodium citrate supplementation prevented terminal differentiation and exhaustion of CAR-T cells, while enhancing their anti-tumor efficacy against human renal clear cell adenocarcinoma (786–0) and glioma (U251) cells, both *in vitro* and *in vivo*. Additionally, the beneficial effects of sodium citrate were also verified in anti-mesothelin (MSLN) CAR-T cells. Further analysis revealed that sodium citrate inhibited calcium signaling, blocked mTOR signaling, and induced metabolic reprogramming in CAR-T cells, thereby preventing exhaustion and enhancing their anti-solid tumor potential. These findings suggest that sodium citrate supplementation during CAR-T cell expansion could improve the efficacy of CAR-T cell therapy in solid tumors by reducing exhaustion and enhancing long-term persistence.

## 2 Materials and methods

### 2.1 Cell lines and cell culture

The human renal clear cell adenocarcinoma 786-0, glioma U251, pancreatic adenocarcinoma Capan-2, triple-negative breast cancer HCC1806 cells were obtained from the American Type Culture Collection (ATCC). Stable cell lines expressing green fluorescent protein (GFP) and firefly luciferase (ffLuc) were generated through lentiviral transduction. CD70 knockout 786-0 cells (CD70 KO 786-0) were generated using the CRISPR/Cas9 system. The 786-0 and 1806 cells were cultured in RPMI 1640 medium (Gibco) supplemented with 10% fetal bovine serum (FBS; Gibco). U251 cells were cultured in DMEM medium (Gibco) with 10% FBS. Capan-2 cells were cultured in McCoy’s 5a medium (Gibco). All cells were maintained in a 37°C incubator with 5% CO_2_.

### 2.2 The generation of CAR-T cells

The generation of anti-CD70 CAR-T cells was carried out following protocols described in our previous studies (34–37). Briefly, human CD70 scFv was obtained through laboratory screening (patent application number: 202410495812.1) (38). The anti-CD70 CAR was constructed by linking the human CD70 scFv with the intracellular CD28/CD3ζ signaling domain, CD8α transmembrane region, CD8α hinge region, and a CD8α signaling peptide. The resulting anti-CD70 CAR was cloned into a pCDH lentiviral vector, and the CAR-expressing plasmid was transfected into HEK293T packaging cells to produce lentiviral particles. Human peripheral blood mononuclear lymphocytes (PBMCs; HYCELLS, hPB050C) were isolated using the Easytep™ Human T Cell Isolation Kit (Stemcell, 17951) and activated with Dynabeads™ Human T-Expander CD3/CD28 beads (Thermo Fisher Scientific, 11141D). Activated T cells were then transduced with the lentiviral particles to generate anti-CD70 CAR-T cells. The generation of anti-MSLN CAR-T cells was established according to our previous research (39). The CAR-T cells were cultured in X-VIVO medium (LONZA) supplemented with IL-2 (100 IU/mL), and the medium was refreshed every 2-3 days to maintain a cell density of 0.5–1×10^6^ cells/mL. Sodium citrate was obtained from Merck (PHR1416) and supplemented in the culture medium 2 days post CAR transduction.

### 2.3 Flow cytometry analysis

T cells or CAR-T cells were collected and labeled with fluorescently conjugated antibodies, followed by a 30-minute incubation. Fluorescein isothiocyanate (FITC)-labeled human CD27 Ligand protein (Acro, CDL-HF249) and PE-labeled human MSLN/Mesothelin protein (Kactus Biosystems, MSL-HM480P) were used to detect the expression of the anti-CD70 and anti-MSLN CAR on CAR-T cells, respectively. FITC anti-CD70 antibody (BioLegend, 355105) was used to assess CD70 antigen expression on tumor cells. To identify CAR-T cell subtypes, APC anti-CD45RA antibody (BioLegend, 304111) and PE anti-CD197/CCR7 antibody (BioLegend, 353203) were utilized. After incubation, cells were washed to remove unbound antibodies, and stained cells were analyzed using a CytoFLEX flow cytometer (Beckman Coulter Life Sciences). Flow cytometry data were processed and analyzed with CytExpert software.

### 2.4 Western blotting

Protein samples were prepared by lysing similar numbers of CAR-T cells from each group with RIPA lysis buffer (Beyotime Biotechnology, P0013B). The lysates were then boiled and denatured before being separated by SDS-PAGE on a 10% acrylamide resolving gel. Following electrophoresis, the proteins were transferred to a polyvinylidene difluoride (PVDF) membrane, blocked in TBS with 5% milk at room temperature for 1 hour, and incubated at 4°C overnight with primary antibodies: anti-Phospho-CamkII (Abclone, AP1386), anti-β-Actin (Abclone, AC026), anti-rpS6 (Cell Signaling, #2217), anti-p-rpS6 (Cell Signaling, #4858) and anti-TBB5 (Abcepta, AW5051). All the primary antibodies were diluted in TBS with 5% BSA at a ratio of 1:1000. After washing, the membrane was incubated with a secondary antibody (diluted in TBST with 5% BSA at a ratio of 1:5000) at room temperature for 1 hour. Protein expression levels were detected using radioautography and quantified using ImageJ.

### 2.5 Cytokine release detection by enzyme-linked immunosorbent assay

Approximately 1×10^6^ anti-CD70 CAR-T cells were resuspended in 500 μL of IL-2-free X-VIVO culture medium and seeded into 24-well flat-bottom plates. After incubating for 24 hours, supernatants were collected for ELISA analysis. ELISA was performed using an ELISA kit (R&D Systems, DIF50C, DTA00D) following the manufacturer’s instructions.

### 2.6 Cytotoxicity detection

The cytotoxicity of CAR-T cells was assessed using the one-lite luciferase assay system (Vazyme, DD1203-02; listed on the manufacturer’s Chinese website) and the real-time cell analysis (RTCA) assay. In the one-lite luciferase assay, T cells or CAR-T cells were co-cultured with tumor cells at different effector-to-target ratios. In the one-lite luciferase assay, after co-culture, 100 μL of luciferase substrate was added to each well and incubated for 5 minutes. Subsequently, the contents of the 96-well plates were transferred to a 96-well ELISA plate, and luminescence intensity, which indicates the viability of tumor cells, was measured using a microplate reader. The cell killing rate was calculated using the formula: Cytotoxicity = (control value - experimental value)/control value × 100%.

In the real-time cell analysis (RTCA) assay, 100 μL of 10 mM L-cysteine was added to each well of an electrode plate, followed by overnight incubation at 37°C. The electrode plate was washed twice with 200 μL/well of sterile water, and 100 μL/well of complete medium was added to zero the analyzer. After emptying the electrode plate, 4×10^4^ tumor cells/well were seeded into the plate, which was placed on a CP96 Real-Time Label-Free Cell Growth Analyzer (USA) for 24 hours to establish a stable cell growth plateau. T cells or CAR-T cells were then added to the electrode plate at the indicated effector-to-target ratios. Data acquisition continued for 80 hours, and data analysis was performed using the acquisition software CP96A. The Cell Index value was recorded, which reflects the number of adherent tumor cells. As CAR-T killed tumor cells, the detached cells resulted in a decrease in the cell index, indicating cytotoxicity.

### 2.7 RNA-sequencing analysis

CAR-T cells were cultured with or without sodium citrate (12 mM) for 10 days. After incubation, the cells were collected, resuspended in TRIzol, and sent for RNA sequencing (RNA-seq), which was performed by Majorbio Bio-Pharm Technology Co., Ltd. (Shanghai, China). Gene Ontology (GO) enrichment analysis was conducted using the Profiler R package. GO terms with P values < 0.05 were considered significantly enriched.

### 2.8 Animal experiments

Animal experiments were conducted at the animal laboratory of Nanjing Normal University in accordance with protocols approved by the Animal Welfare Committee of the university (Approval number: 2020-0047). Immunocompromised B-NDG mice were purchased from Biosetu (Beijing, China) and were ethically bred and maintained in specific pathogen-free (SPF) conditions. Six- to eight-week-old female mice were subcutaneously injected with 5×10^6^ 786-0 cells. When the tumor volume reached approximately 100 mm^3^, the mice were randomly assigned to four treatment groups: PBS group, mock T cell group, control CAR-T cell group, and sodium citrate-pretreated CAR-T cell group, with nine mice in each group. Each group received 200 μL of PBS, 5×10^6^ T cells, 5×10^6^ control CAR-T cells, or 5×10^6^ sodium citrate-pretreated CAR-T cells by tail vein injection. Seven days after injection, three mice from each group were sacrificed for subsequent immunohistochemistry (IHC) and hematoxylin-eosin (H&E) staining experiments. Tumor size and body weight were measured every three days. Tumor volume was calculated using the formula: tumor volume = (length × width^2^)/2. Mice were sacrificed when the tumor size reached approximately 1800 mm^3^.

### 2.9 Immunohistochemistry and hematoxylin-eosin staining

For immunohistochemistry (IHC) staining, tumor tissues were extracted, fixed in 4% paraformaldehyde (PFA), and stained with an anti-CD3 antibody. Tissues from the heart, liver, spleen, lungs, kidneys, and brain were dissected, fixed in 4% PFA, and sent for hematoxylin and eosin (H&E) staining. The staining experiments were carried out by Servicebio Technology Co., Ltd. (Wuhan, China).

### 2.10 Statistical analysis

GraphPad Prism software (version 8.0, GraphPad, Inc., San Diego, CA, USA) was used to analyze data and generate graphs. Data are presented as means ± standard error of the mean (SEM). One-way analysis of variance (ANOVA) and two-way ANOVA with Dunnett’s *post-hoc* test were used for comparisons among different groups. A t-test was applied for comparisons between two groups. A p value of less than 0.05 was considered the threshold for statistical significance in all analyses.

## 3 Results

### 3.1 Tonic signaling induced exhaustion and reduced tumor-killing ability in anti-CD70 CAR-T cells

To specifically target CD70-positive tumor cells, anti-CD70 CAR-T cells were generated by transducing human PBMCs with a lentiviral vector encoding an anti-CD70 CAR construct (**Supplementary Figure S1A**). The expression of the anti-CD70 CAR, as well as CD4 and CD8 on the surface of the CD70-specific CAR-T cells, was confirmed to ensure the quality of the generated CAR-T cells (**Supplementary Figures S1B-C**). Human renal clear cell adenocarcinoma 786-0 cells and glioma U251 cells were selected as target cell lines due to their high expression of CD70 (**Supplementary Figure S1D**). To verify the specificity of the CAR-T cells, we knocked out CD70 in 786-0 cells using the CRISPR/Cas9 system to create CD70 knockout (KO) 786-0 cells (**Supplementary Figure S1E**). Co-culture experiments demonstrated that anti-CD70 CAR-T cells selectively targeted and killed 786-0 cells but showed minimal cytotoxicity against CD70 KO 786-0 cells (**Supplementary Figure S1F**). Additionally, unmodified T cells (mock T) displayed negligible cytotoxicity against cancer cells, further confirming the specificity of the anti-CD70 CAR-T cells (**Supplementary Figures S1G-I**).

**Figure 1:**
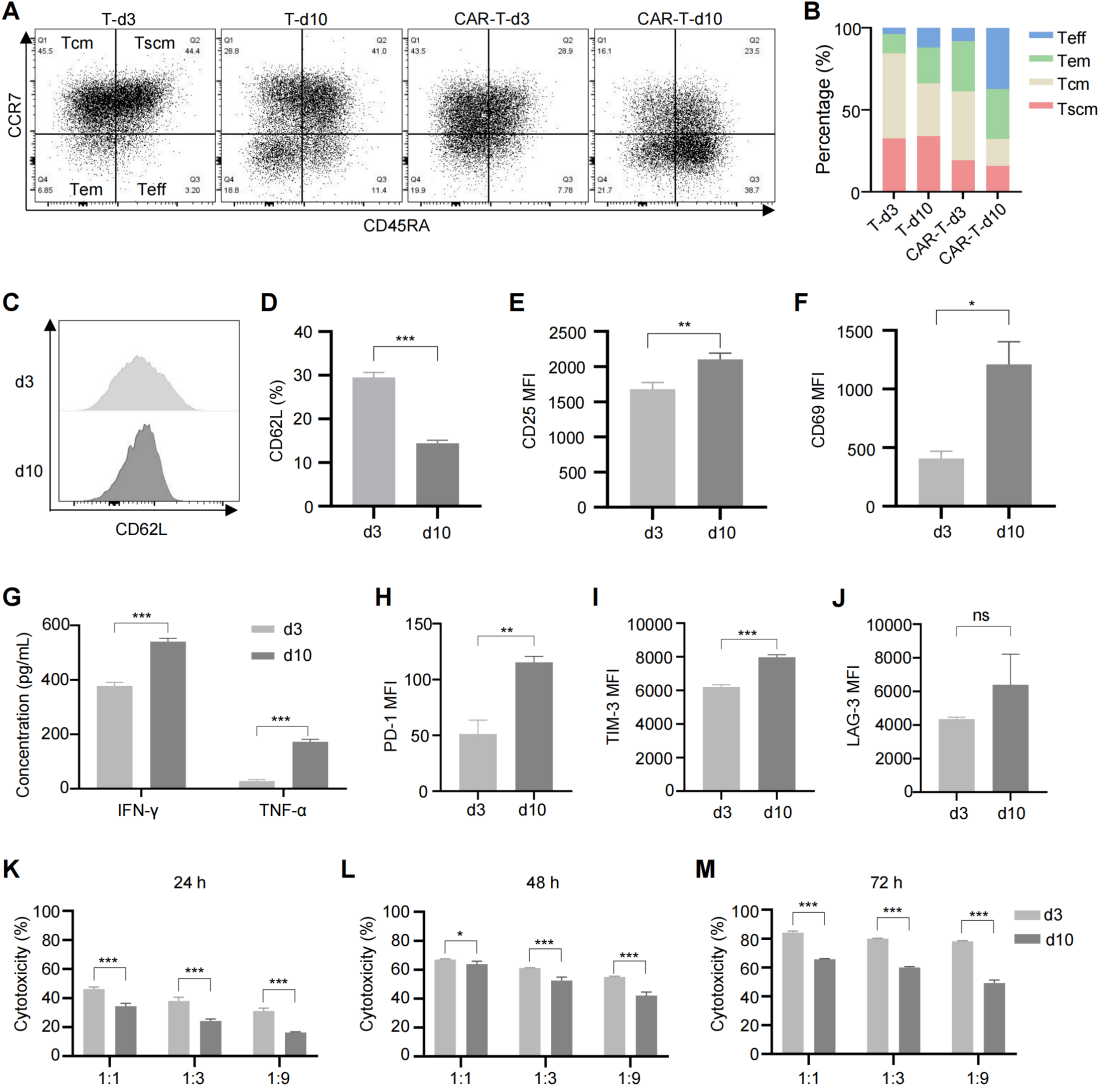
Long-term culture induced CAR-T cell exhaustion, excessive activation and reduced cytotoxicity. **(A)** Representative flow cytometric profile showing the expression of CCR7 and CD45RA on CAR-T cells. **(B)** Proportions of Tscm (CCR7+CD45RA+), Tcm (CCR7+CD45RA-), Tem (CCR7-CD45RA-), and Teff (CCR7-CD45RA+) subsets in anti-CD70 CAR-T cells as measured by flow cytometry. **(C)** Representative flow cytometric profile showing the expression level of CD62L on CAR-T cells. **(D)** Histogram plot showing the percentage of CD62L+ CAR-T cells. **(E, F)** Mean fluorescence intensity (MFI) of CD25 **(E)** and CD69 **(F)** expression on CAR-T cells. **(G)** Levels of TNF-α and IFN-γ released by CAR-T cells at different time points, measured by ELISA. **(H–J)** MFI of exhaustion markers PD-1 **(H)**, TIM-3 **(I)**, and LAG-3 **(J)** in anti-CD70 CAR-T cells. **(K–M)** Cytotoxicity of anti-CD70 CAR-T cells against 786-0 cells after co-culture at the indicated effector-to-target (E:T) ratios for 24 hours **(K)**, 48 hours **(L)**, and 72 hours **(M)**. Data were expressed as mean ± SD from at least 3 independent donors. Statistical significance was determined by *t*-test **(D–F, H–J)** and two-way ANOVA **(G, K–M)**. ns, not significant, *p < 0.05, **p < 0.01, ***p < 0.001.

During *in vitro* expansion of T cells, tonic signaling is progressively activated with increasing culture time. To explore its effects on CAR-T cells, we assessed the phenotypes of CAR-T cells cultured for 3 and 10 days. Compared to day 3, CAR-T cells on day 10 exhibited a reduced proportion of memory cells and a marked shift toward terminal differentiation (**Figures 1A–D**). Consistent with previous studies, we identified memory T cells based on the expression of CD45RA, CCR7, and CD62L (40, 41). While a more comprehensive phenotype includes CD45RO, CD27, and CD28 (42), we focused on the core markers to define this subset in our study. Prolonged culture also led to excessive activation of CAR-T cells, as evidenced by increased expression of activation markers CD25 (**Figure 1E**; **Supplementary Figure S2A**) and CD69 (**Figure 1F**; **Supplementary Figure S2B**), and higher levels of tumor necrosis factor α (TNF-α) and interferon γ (IFN-γ) in the culture medium (**Figure 1G**). Additionally, exhaustion markers including programmed cell death protein 1 (PD-1), T-cell immunoglobulin and mucin-domain containing-3 (TIM-3), and lymphocyte-activation gene 3 (LAG-3) were upregulated (**Figures 1H–J**; **Supplementary Figures S2C-E**), alongside impaired tumor-killing capacity of CAR-T cells at all effector-target ratios (**Figures 1K–M**). These findings confirm that tonic signaling during *in vitro* culture leads to terminal differentiation, excessive activation, exhaustion, and compromised anti-tumor function of CAR-T cells. Improved culturing conditions are needed to generate CAR-T cells with superior therapeutic efficacy.

### 3.2 Sodium citrate inhibited terminal differentiation, activation and exhaustion of anti-CD70 CAR-T cells *in vitro*

**Figure 2:**
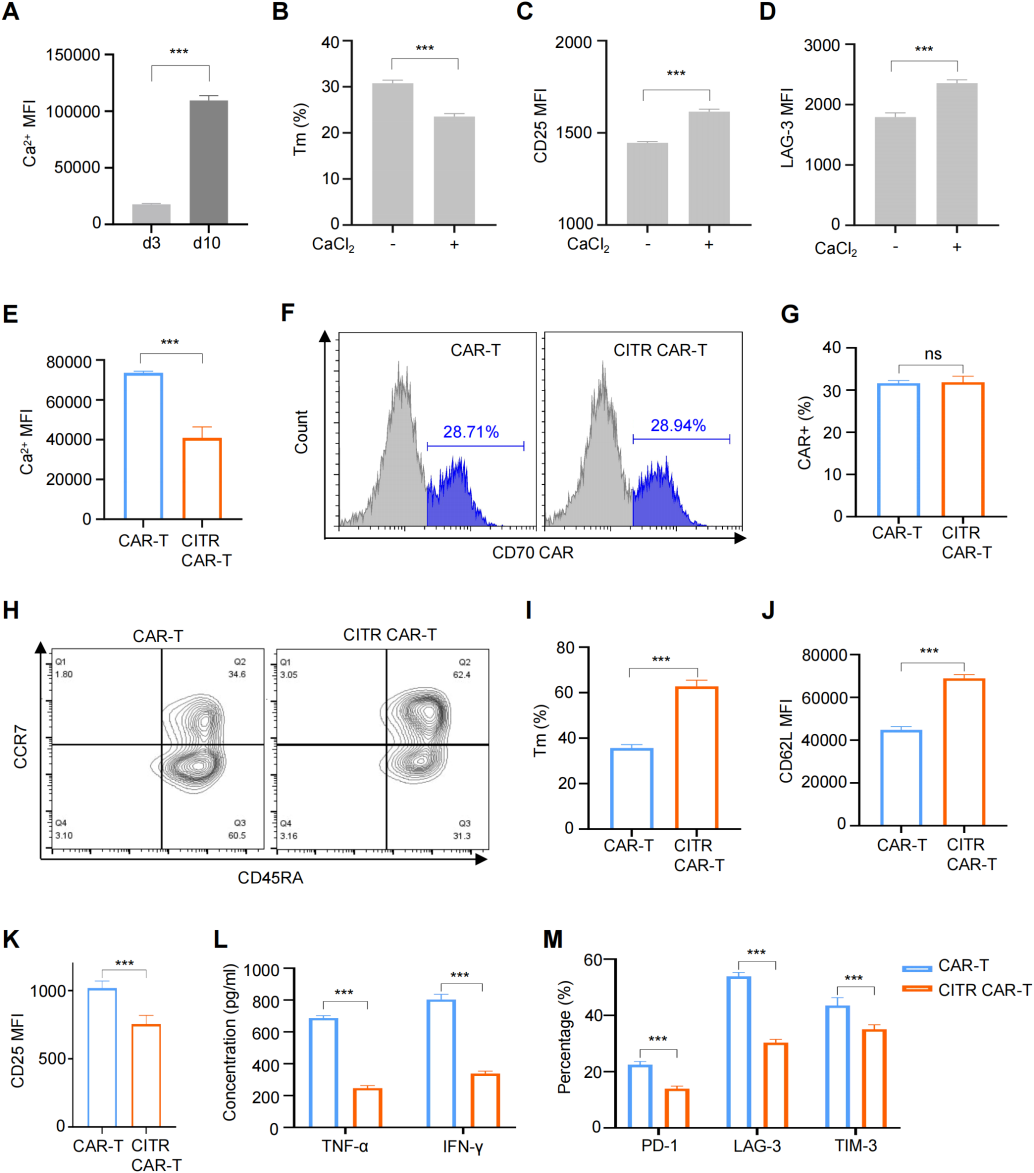
Sodium citrate treatment reduced exhaustion and preserved the memory phenotype of CAR-T cells. **(A)** Intracellular Ca^2+^ levels in CAR-T cells cultured for 3 or 10 days. (B to D) CAR-T cells treated with 6 mM CaCl_2_ for 72 hours, with flow cytometry analysis of memory phenotype **(B)**, excessive activation **(C)**, and exhaustion **(D)** markers. **(E)** Intracellular Ca^2+^ levels in CAR-T cells and CAR-T cells cultured with the supplementation of 12 mM sodium citrate (CITR CAR-T) measured by flow cytometry. **(F, G)** Representative flow cytometric profile **(F)** and histogram **(G)** of CAR expression on CAR-T cells. **(H, I)** Representative flow cytometric profile **(H)** and histogram **(I)** showing the proportion of Tm cells in CAR-T cells (Tscm + Tcm). **(J, K)** Histogram plots showing the MFI of CD62L **(J)** and CD25 **(K)** in CAR-T cells. **(L)** TNF-α and IFN-γ release levels by CAR-T cells measured by ELISA. **(M)** Positive rates of exhaustion markers in CAR-T and CITR CAR-T cells. Results were expressed as mean ± SD from at least 3 independent donors. Statistical significance was determined by *t*-test **(A–E, G, I–K)** and two-way ANOVA **(L, M)**. ns, not significant, ***p < 0.001.

Activated calcium signaling has been linked to T cell exhaustion (23, 28). To assess its role in CAR-T cells, we measured intracellular Ca²^+^ levels in CAR-T cells cultured for 3 and 10 days. Consistent with increased exhaustion and terminal differentiation, intracellular calcium levels were elevated in CAR-T cells cultured for 10 days (**Figure 2A**; **Supplementary Figure S3A**). To further investigate the effects of elevated calcium on CAR-T cells, we treated them with CaCl_2_. As a result, CaCl_2_ treatment reduced the percentage of memory T cells (Tscm + Tcm) and increased the levels of activation and exhaustion markers, confirming the correlation between intracellular calcium and T cell exhaustion (**Figures 2B–D**; **Supplementary Figures S3B-D**).

Given that high intracellular Ca^2+^ levels contribute to T cell exhaustion, we explored calcium signaling inhibition as a strategy to modulate CAR-T cell fate. Among the available inhibitors, sodium citrate was chosen due to its oral availability and minimal safety concerns when applied to CAR-T cells. We next assessed whether sodium citrate supplementation during CAR-T cell culture could mitigate the negative effects of tonic signaling. To identify an optimal sodium citrate concentration—high enough to inhibit intracellular calcium signaling but not adversely affect CAR-T cell proliferation—we first screened a broad range of concentrations (0 to 160 mM) over three days. Sodium citrate concentrations below 10 mM had no significant effect on CAR-T cell proliferation, while concentrations above 20 mM significantly inhibited proliferation (**Supplementary Figure S4A**). We then refined the concentration range (10 to 18 mM) and cultured CAR-T cells for 13 days. A concentration of 12 mM sodium citrate did not significantly impair CAR-T cell proliferation (**Supplementary Figure S4B**), so this concentration was selected for subsequent experiments.

Anti-CD70 CAR-T cells were treated with 12 mM sodium citrate for 7 days, and as expected, intracellular Ca²^+^ levels were reduced in sodium citrate-pretreated CAR-T cells (CITR CAR-T) (**Figure 2E**; **Supplementary Figure S5A**). Sodium citrate treatment did not affect CAR expression (**Figures 2F, G**). Further analysis revealed that sodium citrate-pretreated CAR-T cells had a higher proportion of memory T cells (**Figures 2H–J**) and increased expression of the memory T cell marker L-selectin (CD62L) (**Figure 2J**; **Supplementary Figure S5B**). Additionally, CITR CAR-T cells showed reduced expression of the activation marker CD25 (**Figure 2K**; **Supplementary Figure S5C**) and lower secretion of cytokines TNF-α and IFN-γ without antigen stimulation (**Figure 2L**), suggesting a reduction in excessive activation. Sodium citrate also mitigated CAR-T cell exhaustion, as evidenced by decreased positive rates of exhaustion markers PD-1, TIM-3, and LAG-3 (**Figure 2M**). In conclusion, sodium citrate supplementation improved CAR-T cell characteristics by reducing terminal differentiation, excessive activation, and exhaustion, while promoting a memory phenotype. Further studies to assess *in vitro* and *in vivo* anti-tumor activity are required.

### 3.3 Sodium citrate-pretreated CAR-T cells showed enhanced *in vitro* tumor-killing ability

**Figure 3:**
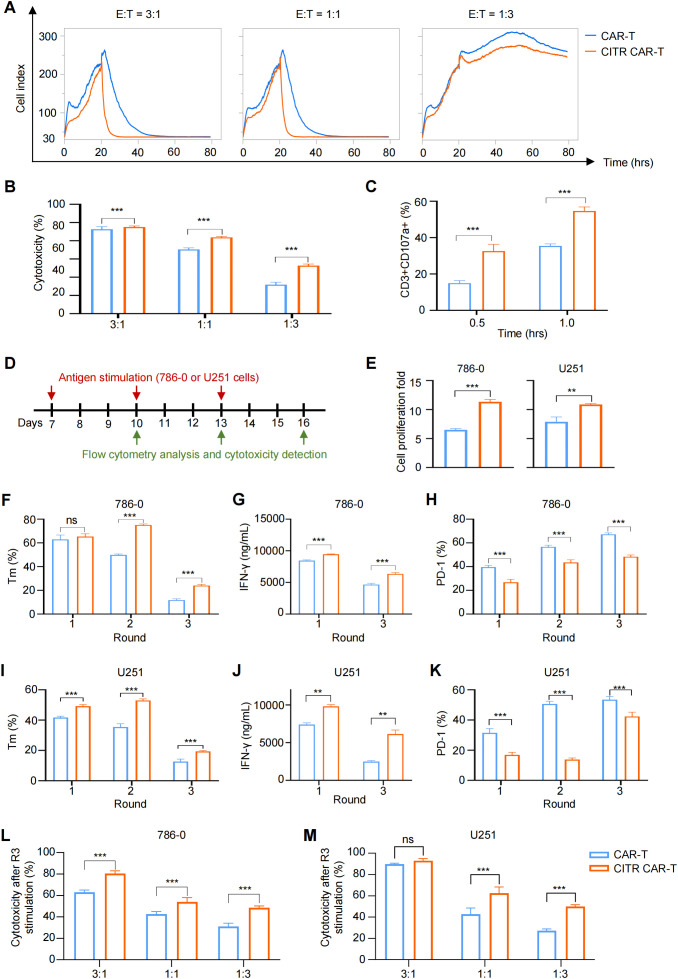
Sodium citrate pretreatment enhanced the anti-tumor cytotoxicity of CAR-T cells. **(A)** Real-time cell analysis was performed to monitor the cytotoxicity of CAR-T cells. Cell index value reflects the viability of tumor cells. 786-0 cells were allowed to attach for 24 hours, and mock T or CAR-T cells were then added and co-cultured with 786-0 cells until 80 hours. **(B)** Cytotoxicity of CAR-T or mock T cells co-cultured with 786-0 cells at E:T ratios of 3:1, 1:1, and 1:3 for 24 hours, measured by One-Lite Luciferase Assay. **(C)** CD107a expression in CAR-T cells after 0.5 and 1 hour of co-culture with 786-0 cells. **(D)** Schematic of multiple rounds of antigen stimulation. **(E–K)** Proliferation **(E)**, memory phenotype (CCR7+) **(F, I)**, IFN-γ secretion **(G, J)**, and exhaustion markers **(H, K)** of CAR-T cells after each round of stimulation. **(L, M)** Cytotoxicity of CAR-T cells after three rounds of stimulation, co-cultured with 786-0 **(L)** or U251 **(M)** cells at indicated E:T ratios, assessed by One-Lite Luciferase Assay. Results were expressed as mean ± SD from at least 3 independent donors and statistical significance was determined by two-way ANOVA **(B, C, F–M)** and *t*-test **(E)**. ns, not significant, **p < 0.01, ***p < 0.001.

To evaluate the effect of sodium citrate pretreatment on the tumor-killing ability of CAR-T cells, we co-cultured CAR-T cells with tumor cells and assessed their interactions using real-time cell analysis (RTCA) and a luciferase reporter assay. In both assays, CITR CAR-T cells exhibited significantly higher cytotoxicity compared to untreated CAR-T cells, confirming the pro-killing effect of sodium citrate on CAR-T cells (**Figures 3A, B**). Furthermore, the expression of CD107a, a marker positively correlated with T cell degranulation and cytotoxicity (43), was higher in CITR CAR-T cells upon co-culture with 786-0 cells (**Figure 3C**).

Repetitive antigen stimulation in the tumor microenvironment is a key factor responsible for T cell dysfunction. To assess the long-term effects, we repeatedly stimulated CAR-T cells with 786-0 and U251 cells and analyzed their features and functions (**Figure 3D**). CITR CAR-T cells demonstrated significantly higher proliferative capacity than untreated CAR-T cells in response to repeated antigen stimulation (**Figure 3E**). Moreover, after multiple rounds of stimulation, CITR CAR-T cells displayed a high proportion of memory T cells, increased cytokine secretion, and reduced exhaustion in both 786-0 (**Figures 3F–H**) and U251 stimulation models (**Figures 3I–K**). In terms of anti-tumor activity, CITR CAR-T cells exhibited more effective tumor cell killing than untreated CAR-T cells after three rounds of antigen stimulation, as shown by the luciferase reporter assay (**Figures 3L, M**). Thus, sodium citrate pretreatment enhanced the anti-tumor efficacy of CAR-T cells in both short-term and long-term models *in vitro*.

### 3.4 Sodium citrate supplementation enhanced the *in vivo* anti-tumor activity of CAR-T cells and inhibited cancer recurrence

**Figure 4:**
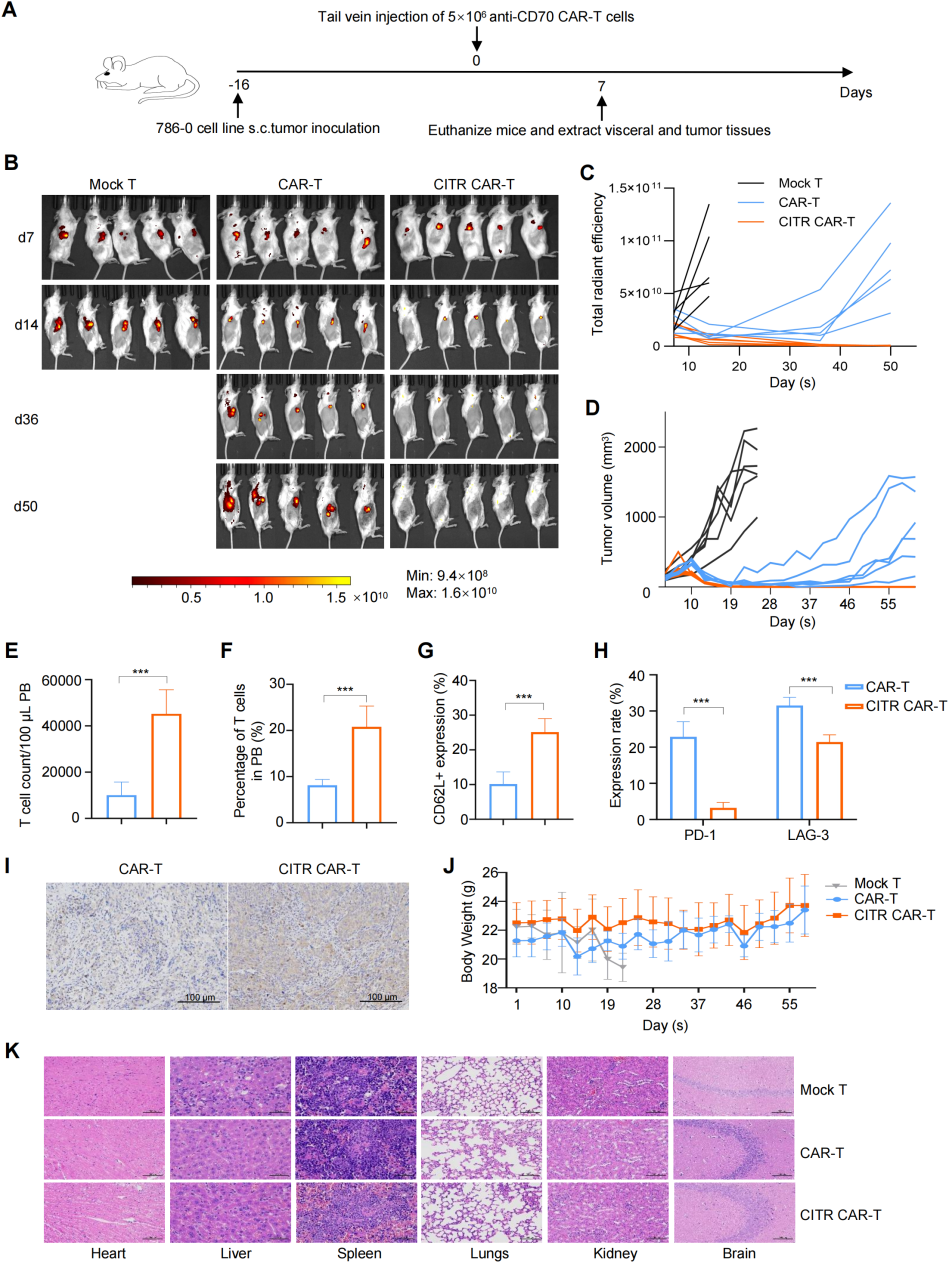
Sodium citrate-pretreated CAR-T cells exhibited enhanced anti-solid tumor activity and reduce cancer relapse in mice. **(A)** Experimental setup: 786-0 tumor cells were subcutaneously injected into NKG mice on day 0. PBS, mock T cells, untreated anti-CD70 CAR-T cells, or sodium citrate-pretreated anti-CD70 CAR-T cells were injected via tail vein on day 16. **(B, C)** BLI images **(B)** and tumor growth quantification **(C)** at indicated time points (n = 5). **(D)** Tumor volumes over time for each group (n = 6). **(E, F)** Flow cytometry analysis of CAR-T cell number **(E)** and percentage **(F)** in peripheral blood at day 6 (n = 6). **(G)** CD62L expression in CAR-T cells from peripheral blood (n = 6). **(H)** PD-1 and LAG-3 expression in CAR-T cells from peripheral blood (n = 6). **(I)** Representative IHC staining for CD3 in tumor tissues from NKG mice treated with untreated or sodium citrate-pretreated CAR-T cells. Scale bar = 100 μm. **(J)** Body weight changes of mice throughout the experiment (n = 6). **(K)** Representative H&E staining of heart, liver, spleen, lungs, kidneys and brain from mock T, untreated CAR-T, and sodium citrate-pretreated CAR-T groups. Scale bar = 100 μm. Results were expressed as mean ± SD. Statistical significance was determined by *t*-test **(E–G)** and two-way ANOVA **(H)**. ***p < 0.001.

To assess the *in vivo* tumor-killing ability of CITR CAR-T cells, we established a mouse subcutaneous tumor model using 786-0 tumor cells and injected mock T, untreated, or CITR CAR-T cells via the tail vein (**Figure 4A**). Both CAR-T and CITR CAR-T cells effectively inhibited tumor growth during the first 16 days post-injection. However, mice treated with CITR CAR-T cells showed a significantly lower tumor burden, as indicated by bioluminescence imaging (BLI) radiance (**Figures 4B, C**) and tumor volume measurements (**Figure 4D**). While local recurrence occurred in all mice in the untreated CAR-T cell group, tumors in the CITR CAR-T cell group remained inhibited throughout the experiment, suggesting superior persistence of CITR CAR-T cells *in vivo*.

Six days after CAR-T cell injection, peripheral blood was collected from mice for flow cytometry analysis. The results revealed that the number and proportion of CAR-T cells were significantly higher in the CITR CAR-T group compared to the untreated CAR-T group (**Figures 4E, F**). Further analysis showed increased expression of the memory T cell marker CD62L (**Figure 4G**), reduced expression of exhaustion markers PD-1 and LAG-3 in CITR CAR-T cells (**Figure 4H**), and enhanced CAR-T cell infiltration in the tumors of the CITR CAR-T group (**Figure 4I**).

To evaluate the safety of CITR CAR-T cells, we monitored the body weight of mice throughout the study. Mice in the CITR CAR-T group maintained relatively higher body weights compared to those in the other groups (**Figure 4J**). Additionally, H&E staining of major organs, including heart, liver, spleen, lung, kidney and brain) revealed no significant organ damage in the CITR CAR-T group compared to the mock T cells (**Figure 4K**). In summary, sodium citrate supplementation enhanced the anti-tumor efficacy, particularly the anti-recurrence ability, of CAR-T cells, without compromising their safety.

### 3.5 Sodium citrate reduced exhaustion and increased anti-tumor capacity of anti-MSLN CAR-T cells

**Figure 5:**
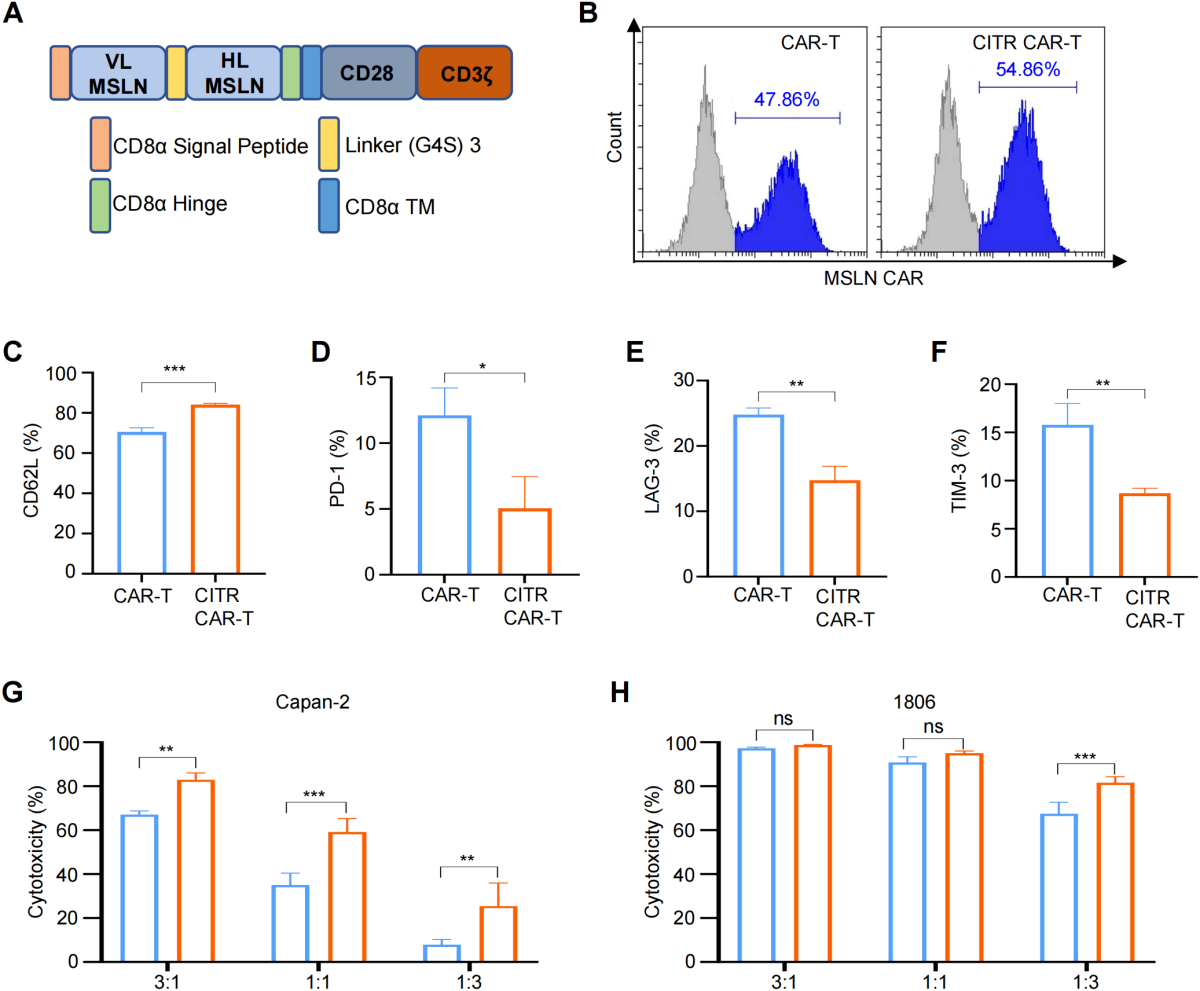
Sodium citrate alleviated exhaustion and enhanced anti-tumor activity of anti-MSLN CAR-T cells. **(A)** Schematic representation of the anti-MSLN CAR construct. **(B)** Representative flow cytometric analysis of anti-MSLN CAR expression in CAR-T cells with or without sodium citrate treatment. **(C–F)** Positive rates of CD62L **(C)**, PD-1 **(D)**, LAG-3 **(E)**, and TIM-3 **(F)** in CAR-T and CITR CAR-T cells. **(G, H)** Cytotoxicity of CAR-T and CITR CAR-T cells co-cultured with Capan-2 **(G)** and 1806 **(H)** cells at indicated E:T ratios for 6 hours, assessed by one-lite luciferase assay system. Results were expressed as mean ± SD from at least 3 independent donors. Statistical significance was determined by *t*-test **(C–F)** and two-way ANOVA **(G, H)**. ns, not significant, *p < 0.05, **p < 0.01, ***p < 0.001.

In previous work, we developed anti-MSLN CAR-T cells for the treatment of mesothelin-positive cancers (**Figure 5A**) (39). To assess whether the beneficial effects of sodium citrate observed in anti-CD70 CAR-T cells could be extended to anti-MSLN CAR-T cells, we treated these cells with sodium citrate. Supplementation with sodium citrate did not significantly affect the CAR-positive rate (**Figure 5B**). As seen in anti-CD70 CAR-T cells, sodium citrate treatment upregulated CD62L expression in anti-MSLN CAR-T cells (**Figure 5C**) and reduced the expression of exhaustion markers (**Figures 5D–F**). Furthermore, when sodium citrate-pretreated CAR-T cells were co-cultured with Capan-2 and HCC1806 cells, they exhibited enhanced tumor-killing activity, particularly at lower E:T ratios (**Figures 5G, H**). Together, the role of sodium citrate in reducing exhaustion and enhancing anti-tumor activity is verified in both anti-CD70 and anti-MSLN CAR-T cells.

### 3.6 Sodium citrate impeded CAR-T cell terminal differentiation via inhibition of mTOR and glycolysis pathways

**Figure 6:**
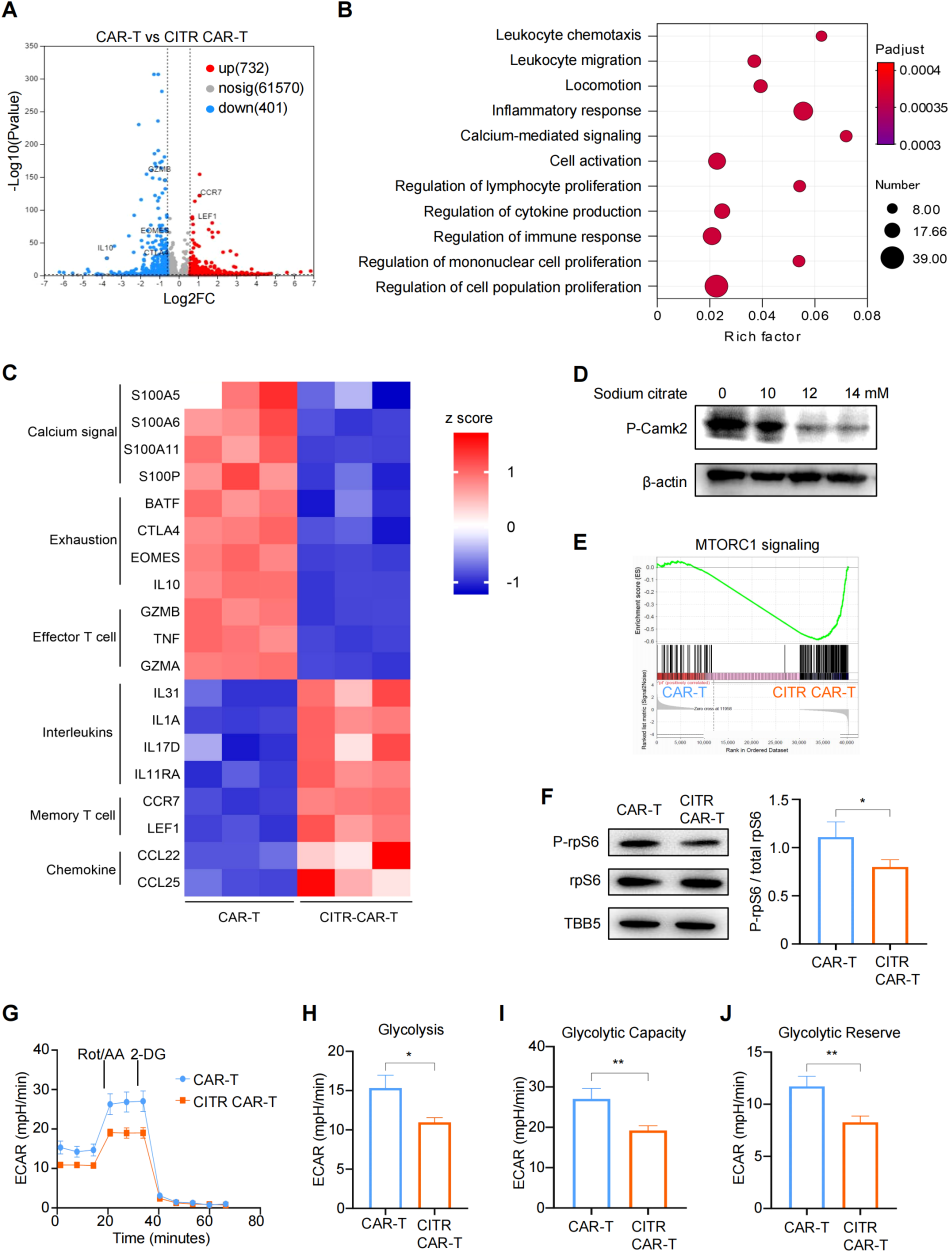
Sodium citrate blocked mTOR signaling and glycolysis pathways through calcium inhibition in CAR-T cells. **(A)** Volcano plot showing differentially expressed genes (DEGs) in sodium citrate-pretreated CAR-T cells compared with untreated CAR-T cells, with significance defined by fold change >2 or <0.5 and -Log10Pvalue > 2. **(B)** Gene ontology (GO) term enrichment analysis of DEGs between CAR-T and CITR CAR-T cells. **(C)** Heatmap of DEGs involved in calcium signaling and T cell function. **(D)** Western blot analysis of Camk2 phosphorylation in CAR-T cells stimulated with PMA and ionomycin, treated with PBS or sodium citrate (10 mM, 12 mM, 14 mM) for 3 days. **(E)** Gene set enrichment analysis (GSEA) plot showing mTORC1 signaling enrichment in CITR CAR-T cells. **(F)** (left) Western blot analysis of rpS6 and P-rpS6 levels in untreated and sodium citrate-pretreated CAR-T cells. (right) The relative level of P-rpS6 to total rpS6 was quantified using ImageJ. **(G–J)** Extracellular acidification rate (ECAR) measurement in CAR-T cells with or without sodium citrate pretreatment, with calculated values for glycolysis **(H)**, glycolytic capacity **(I)**, and glycolytic reserve **(J)**. Results were expressed as mean ± SD from at least 3 independent donors. Statistical significance was determined by *t*-test. *p < 0.05, **p < 0.01.

To explore the mechanisms underlying the sodium citrate-induced reduction in CAR-T cell exhaustion, we collected anti-CD70 CAR-T cells cultured with or without sodium citrate supplementation and performed RNA sequencing (RNA-seq) to compare the gene expression profiles and identified differentially expressed genes (DEGs) (**Figure 6A**). Gene ontology (GO) enrichment analysis of these DEGs revealed that several immune response-related pathways, as well as the calcium-mediated signaling pathway, were affected by sodium citrate (**Figure 6B**). Additionally, markers of exhaustion and effector T cells, such as eomesodermin (EOMES), interleukin-10 (IL10), cytotoxic T-lymphocyte-associated protein 4 (CTLA4), and granzyme B (GZMB), were also reduced. In contrast, genes associated with memory T cells, including lymphoid enhancer-binding factor 1 (LEF1) and C-C motif chemokine receptor 7 (CCR7), were upregulated in CITR CAR-T cells (**Figure 6C**).

We next assessed the phosphorylation levels of calcium/calmodulin-dependent protein kinase II (CamkII) by western blotting and confirmed that sodium citrate inhibited calcium signaling in a dose-dependent manner (**Figure 6D**). To further investigate the functional differences between CITR CAR-T cells and untreated CAR-T cells, we performed gene set enrichment analysis (GSEA). This analysis revealed that genes related to the mechanistic target of rapamycin complex 1 (mTORC1) signaling pathway were significantly downregulated in sodium citrate-treated CAR-T cells (**Figure 6E**). Consistently, western blotting results showed reduced phosphorylation of ribosomal protein S6 (rpS6), a marker of mTORC1 activity (**Figure 6F**).

**Figure 7:**
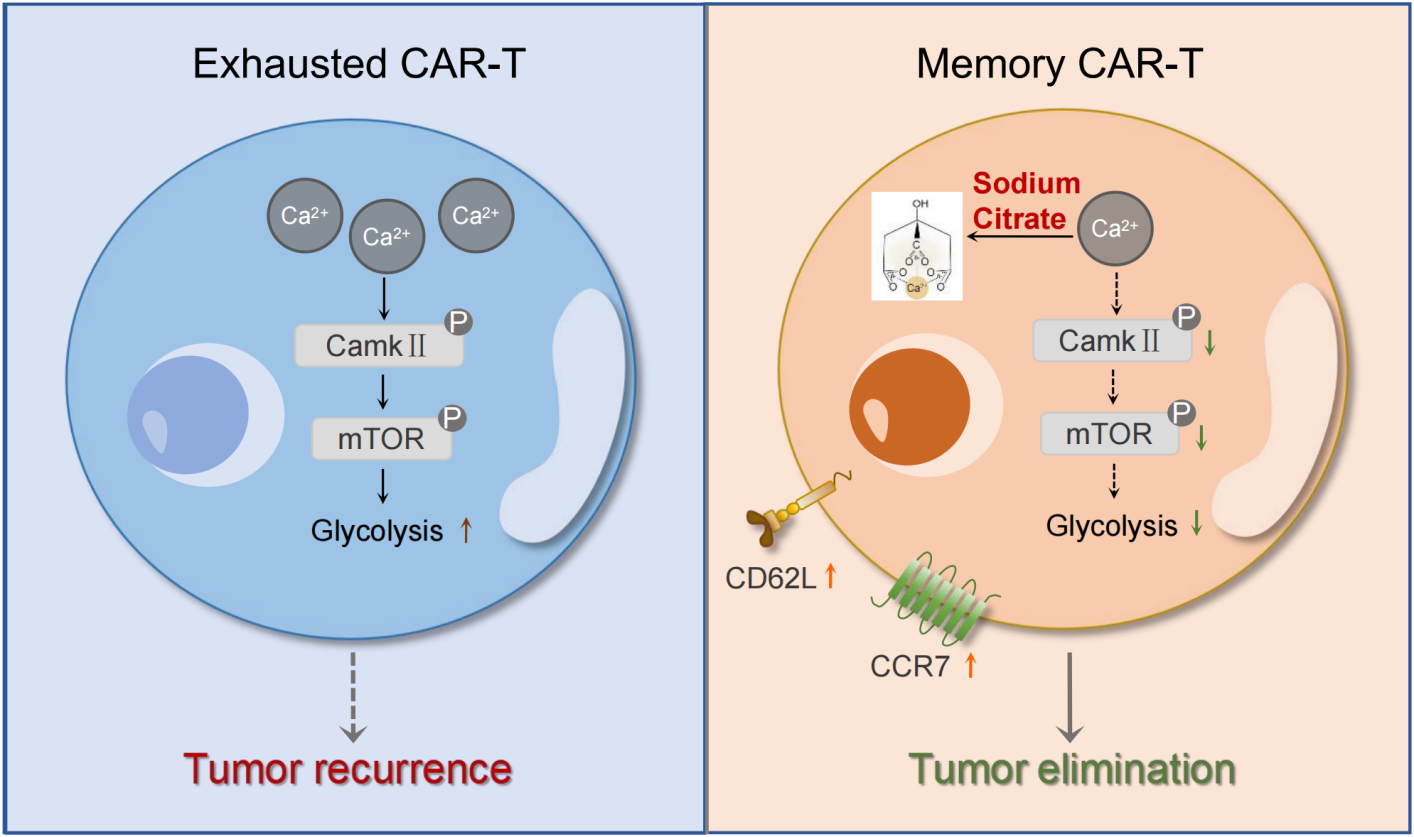
Mechanism diagram of the effects of sodium citrate on CAR-T cells. Sodium citrate chelates Ca^2+^, reducing intracellular Ca^2+^ levels. This inhibition of calcium signaling decreases CamkII phosphorylation, which in turn blocks mTORC1 activity and glycolysis. These effects prevent T cell exhaustion and promote the generation of memory T cells, enhancing CAR-T cell function.

Since inhibition of the mTOR pathway has been shown to decrease glycolysis and prevent T cell terminal differentiation and exhaustion (44), we used Seahorse assays to assess the glycolytic activity of CAR-T cells. CITR CAR-T cells exhibited a significant reduction in glycolysis, glycolytic capacity, and glycolytic reserve compared to untreated CAR-T cells (**Figures 5G–J**). Together, these findings suggest that sodium citrate reduces the calcium level in CAR-T cells and inhibits calcium signaling, which in turn blocks mTOR signaling and glycolysis in CAR-T cells, thus reducing their exhaustion and promoting the formation of memory T cells (**Figure 7**). Supplementing sodium citrate in the culture medium could therefore enhance CAR-T cell functionality.

## 4 Discussion

Numerous clinical cases have demonstrated the effectiveness of CAR-T cell therapy in hematological malignancies, but challenges remain in treating solid tumors. One major obstacle is T cell exhaustion, induced by the immunosuppressive tumor microenvironment, which limits the efficacy of CAR-T therapy. Evidence has shown that T cell exhaustion reduces the tumor-killing capacity of CAR-T cells (11, 21). Tonic signaling in CARs, which leads to T cell terminal differentiation and exhaustion, further impairs CAR-T efficacy (45). Our findings show that tonic signaling induces terminal differentiation, excessive activation, exhaustion and reduced tumor-killing ability in anti-CD70 CAR-T cells during *in vitro* culture (**Figure 1**). Addressing tonic signaling in CAR-T cells is thus considered a promising strategy to overcome T cell exhaustion and improve the clinical effectiveness of CAR-T therapy for solid tumors (46).

The calcium signaling pathway plays a crucial role in regulating immune responses, influencing cytokine release, differentiation, and metabolism in T cells (47, 48). Previous study found that calcium signaling was activated by tonic signaling in CAR-T cells, while a store-operated calcium entry (SOCE) inhibitor BTP-2, but not the calcium chelator BAPTA-AM enhanced the anti-leukemia efficacy of CAR-T cells (23). Consistently, we observed that tonic signaling increased the level of Ca^2+^ in CAR-T cells, and we further verified that inhibition of calcium signaling could improve the cytotoxicity of CAR-T cells against solid tumors. However, different from the previous study, we found that the calcium chelator, sodium citrate, displayed beneficial effects on CAR-T cells. Sodium citrate is a widely used and safe organic acid salt, which is readily accessible and cost-effective (49, 50). In our study, we identified an optimal sodium citrate concentration for treating CAR-T cells and employed a simple *in vitro* treatment method. Sodium citrate pretreatment promoted the expression of memory T cell markers and reduced the expression of activation and exhaustion markers in both anti-CD70 and anti-MSLN CAR-T cells, as well as the anti-solid tumor activity. Particularly, sodium citrate-pretreated CAR-T (CITR CAR-T) cells outperformed untreated CAR-T cells in persistence *in vivo*. Mice infused with untreated CAR-T cells experienced tumor recurrence, while mice treated with CITR CAR-T cells showed sustained tumor inhibition throughout the experiment. These findings indicate that sodium citrate supplementation could advance the clinical application of CAR-T cells for treating solid tumors.

Inhibition of the mTOR pathway and glycolysis has been shown to promote the generation of memory T cells and reduce T cell exhaustion (51–53). Previous studies have demonstrated that citrate suppresses tumor growth by inhibiting calcium signaling, mTOR and glycolysis pathways (30, 54). Consistent with these findings, our study revealed that both RNA-seq and Western blot analysis confirmed the suppression of calcium signaling and the mTOR pathway in CITR CAR-T cells. Furthermore, seahorse assays indicated that glycolysis in CAR-T cells was inhibited following sodium citrate treatment. Based on these results, we propose that sodium citrate pretreatment reduces intracellular Ca^2+^ levels in CAR-T cells, leading to the inhibition of CamkII phosphorylation. This, in turn, suppresses mTORC1 signaling and glycolysis, thereby promoting the differentiation of memory T cells and mitigating exhaustion. This mechanism enhances the anti-tumor function of CAR-T cells in solid tumors, particularly improving the persistence of CAR-T therapy.

Since a cell-derived xenograft (CDX) model was used in this study, it may not fully replicate the tumor microenvironment observed in patients. Future studies should consider using patient-derived xenograft (PDX) models, which may provide more clinically relevant insights. Additionally, given that sodium citrate has direct tumoricidal effects, a combination therapy of CAR-T cells with sodium citrate could be explored to enhance the overall anti-tumor response.

In conclusion, our findings demonstrate that sodium citrate plays a crucial role in preventing CAR-T cell exhaustion and terminal differentiation. By inhibiting calcium signaling, mTOR activity, and glycolysis, sodium citrate enhances CAR-T cell persistence and promotes the development of memory T cells, which improves their anti-tumor efficacy. These results provide a promising strategy for enhancing the clinical application of CAR-T cell therapy in treating solid tumors.

## Data Availability

The data presented in the study are deposited in the NCBI repository, accession number PRJNA1233279.
