# Supplementary material for: Sodium citrate pretreatment enhances CAR-T cell persistence and anti-tumor efficacy through inhibition of calcium signaling
Source: Front Immunol. 2025 Mar 17;16:1540754. doi: 10.3389/fimmu.2025.1540754 (PMC11955688; doi:10.3389/fimmu.2025.1540754)
Supplement: Supplementary file 1 [file DataSheet1.docx]

**Supplementary Materials**

**
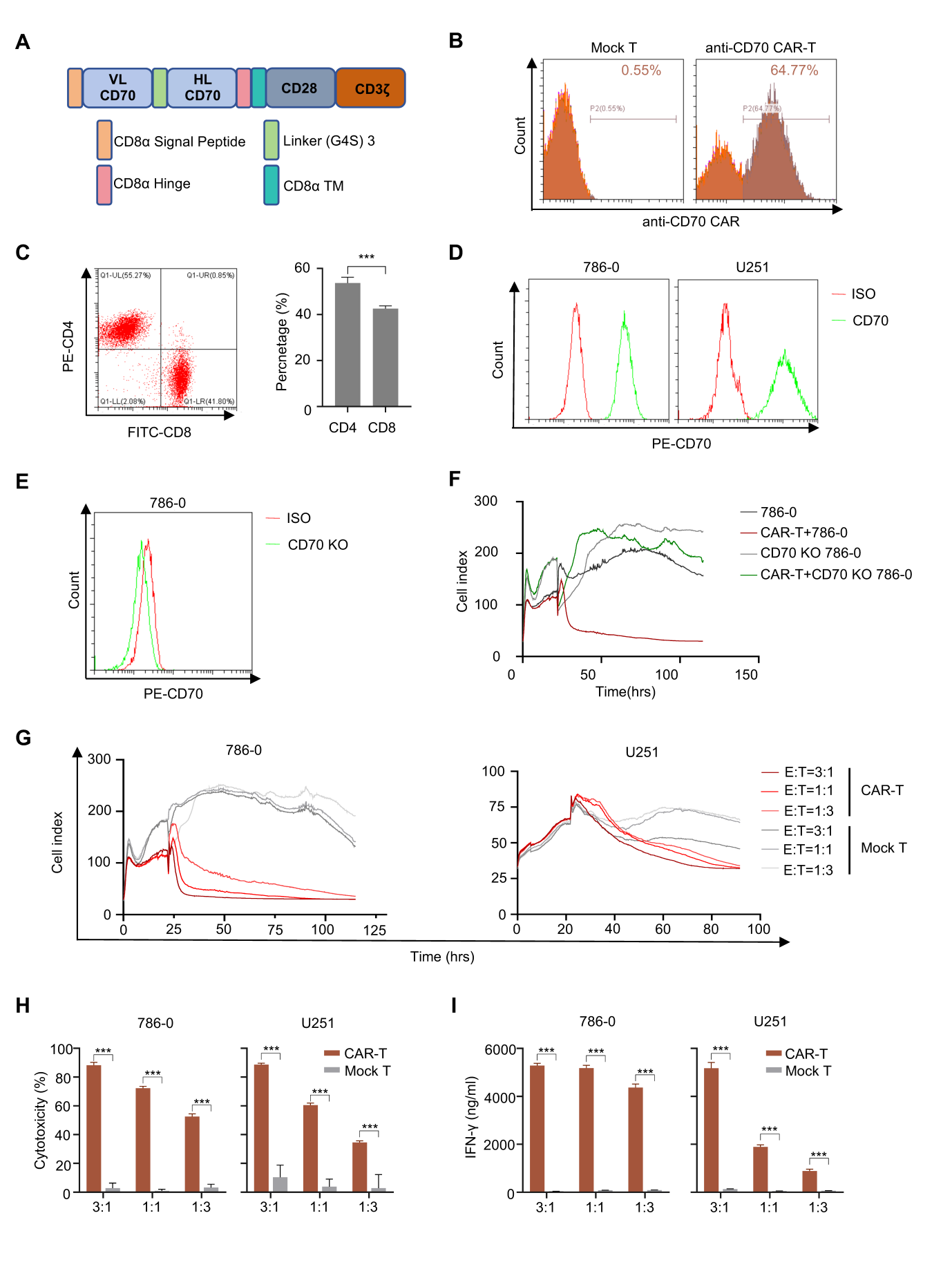
**

**Figure S1. Generation and functional characterization of anti-CD70 CAR-T cells.** (A) Schematic diagram of the anti-CD70 CAR structure. (B) Flow cytometry analysis showing the percentage of CAR-positive T cells. (C) Flow cytometric profiles (left) and histogram plots (right) of CD4+ and CD8+ T cells in CAR-T cell populations. (D-E) Flow cytometry analysis showing CD70 expression on 786-0 and U251 tumor cells (D), and CD70 knockout 786-0 tumor cells (E). (F) Cell index values in co-cultures of CAR-T cells with 786-0 or CD70 KO 786-0 cells. (G-H) Cytotoxicity of CAR-T cells against 786-0 or U251 cells, assessed by RTCA (G) and one-lite luciferase assay system (H) at various E:T ratios. (I) IFN-γ secretion levels in supernatants measured by ELISA. Results were expressed as mean ± SD from at least 3 independent donors. Statistical significance was determined by t-test (C) and two-way ANOVA (H and I). ***p < 0.001.


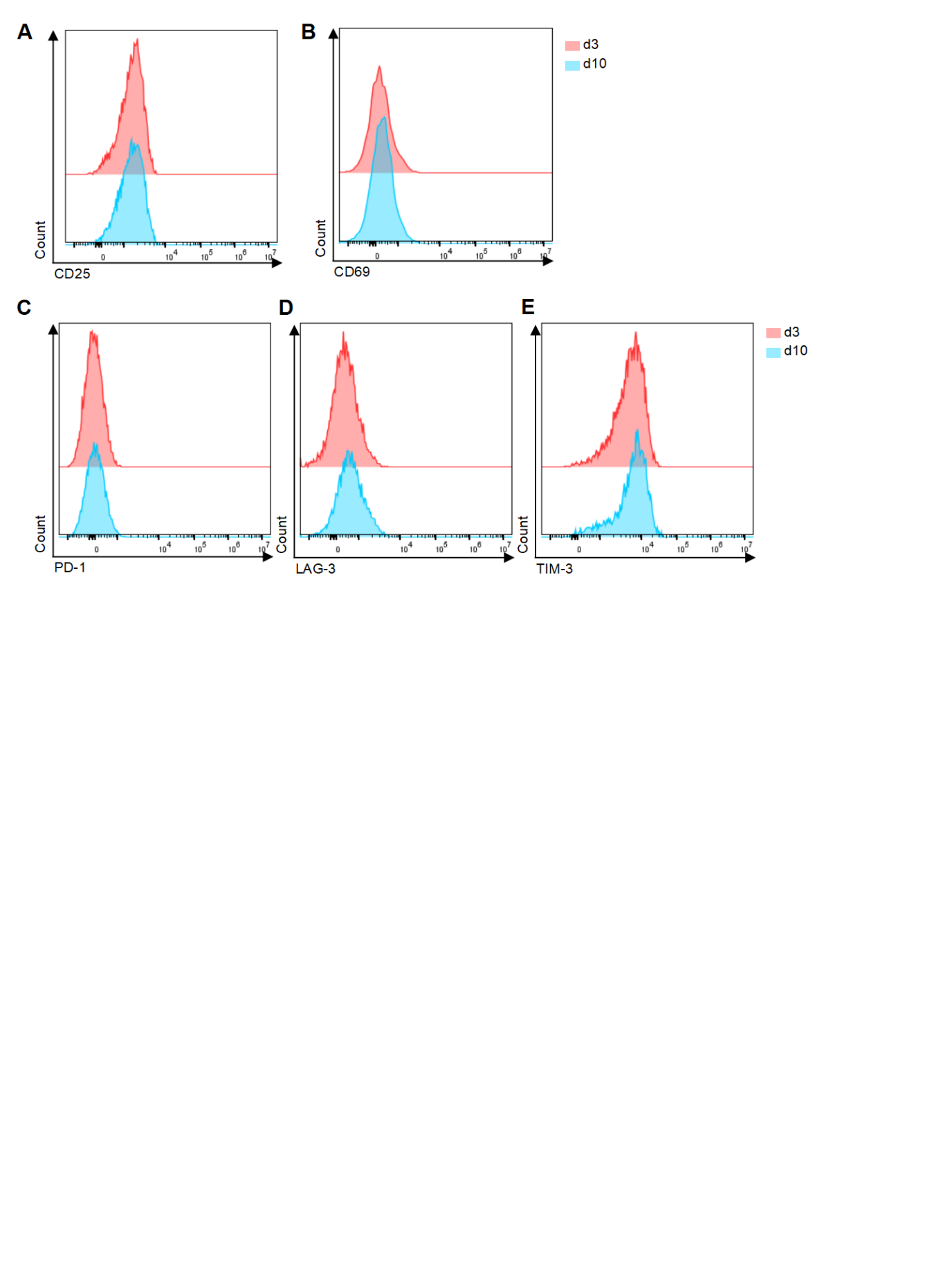


**Figure S2. Tonic signaling caused excessive activation and exhaustion in CAR-T cells.** (A-B) Representative histograms showing the expression levels of CD25 (A) and CD69 (B) in CAR-T cells on day 3 and day 10. (C-E) Representative histograms showing the expression levels of PD-1 (C), LAG-3 (D), and TIM-3 (E) in CAR-T cells on day 3 and day 10.


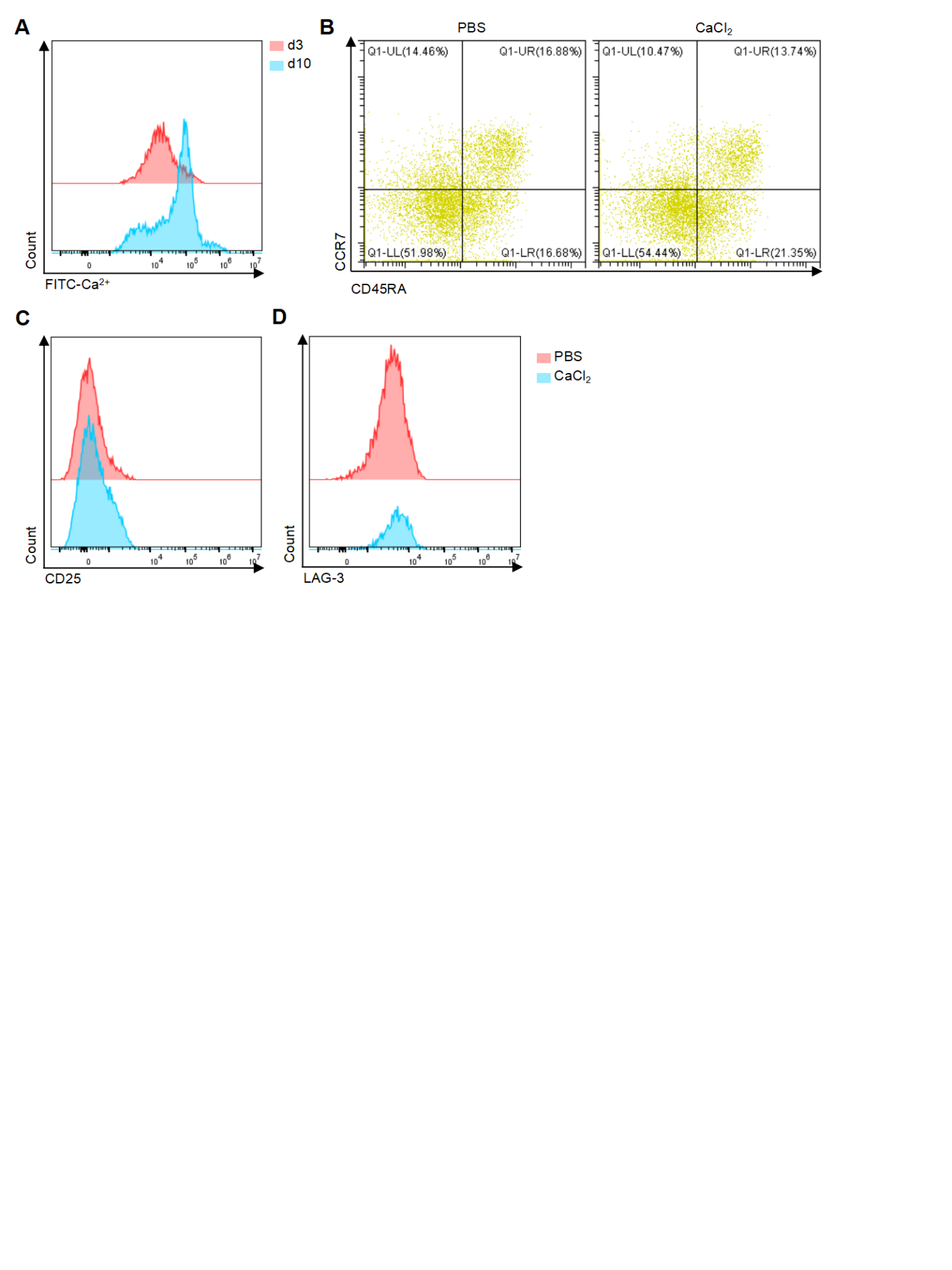


**Figure S3. Increased calcium signaling decreased the memory phenotype and induced excessive activation and exhaustion in CAR-T cells** (A) Intracellular Ca^2+^ levels in CAR-T cells cultured for 3 or 10 days. (B) Representative flow cytometric profiles showing the expression of CCR7 and CD45RA in CAR-T cells treated with PBS or CaCl_2_. (C-D) Representative histograms showing the expression levels of CD25 (C) and LAG-3 (D) in CAR-T cells treated with PBS or CaCl_2_.

**

Figure S4. Dose-dependent effect of sodium citrate on proliferation of CAR-T cells during *in vitro* culture.** (A) Total cell number of CAR-T cells treated with 0-160 mM sodium citrate for 24, 48, and 72 hours. (B) CAR-T cells treated with 0, 10, 12, 14, 16, and 18 mM sodium citrate for 13 days. Total cell numbers were recorded at the indicated time points. Results are expressed as mean ± SD from at least 3 independent donors. Statistical significance was determined by two-way ANOVA. ns, not significant; ***p < 0.001.


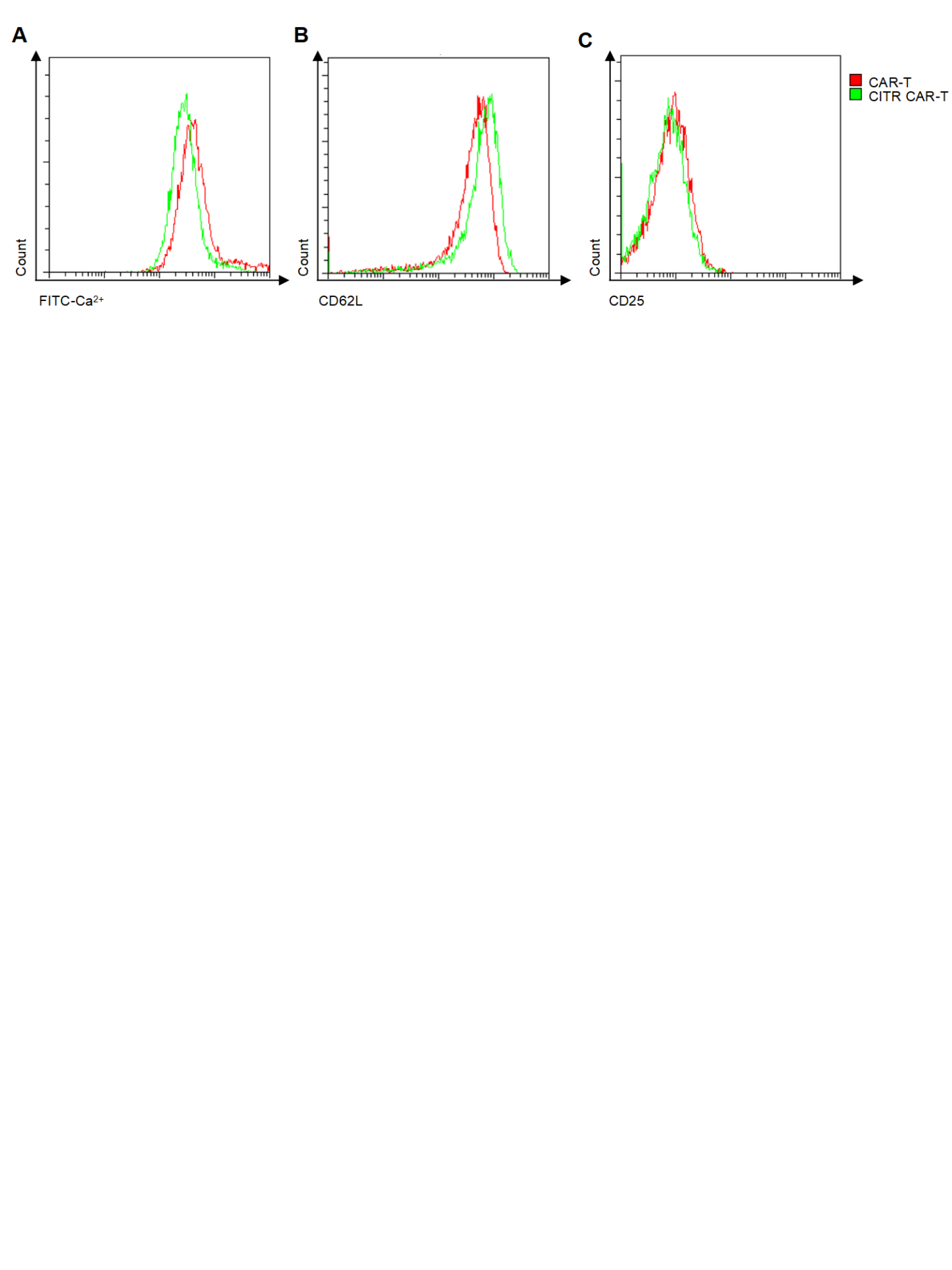


**Figure S5. Sodium citrate inhibited calcium signaling and increased the memory phenotype of CAR-T cells.** (A) Representative histogram showing intracellular Ca^2+^ levels in CAR-T and CITR CAR-T cells. (B-C) Representative histograms showing the expression levels of CD62L (B) and CD25 (C) in CAR-T and CITR CAR-T cells.
